# Elimination of Intracardiac Shunting Provides Stable Gas Anesthesia in Tortoises

**DOI:** 10.1038/s41598-018-35588-w

**Published:** 2018-11-20

**Authors:** Eva Maria Greunz, Catherine Williams, Steffen Ringgaard, Kasper Hansen, Tobias Wang, Mads Frost Bertelsen

**Affiliations:** 1 Center for Zoo and Wild Animal Health, Copenhagen Zoo, Roskildevej 38, 2000 Frederiksberg, Denmark; 2 Section of Zoophysiology, Department of Bioscience, Aarhus University, 8000 Aarhus C, Denmark; 3 MR Research Center, Department of Clinical Medicine, Aarhus University Hospital, 8200 Aarhus N, Denmark; 4 Aarhus Institute of Advanced Sciences, Aarhus University, 8000 Aarhus C, Denmark

## Abstract

Inhalant anesthesia is challenging in chelonians due to a great capacity for breath-holding and an incomplete separation of the cardiac ventricle. Deoxygenated blood can recirculate back into systemic circulation by bypassing the lung in a process referred to as intracardiac right to left (R-L) shunting. Via electrocardiogram gated magnetic resonance imaging, a novel modality to investigate arterial flows in reptiles, intracardiac shunting and its elimination via atropine during gas anesthesia in tortoises (*Chelonoidis carbonaria*) was demonstrated. The great vessels of the heart were visualized confirming that after shunt-elimination, the flow (mean ± sd) in the pulmonary arteries increased significantly (54.6 ± 9.5 mL min^−1^ kg^−1^ vs 10.8 ± 3.4 mL min^−1^ kg^−1^; P < 0.008). Consequently, animals required significantly lower concentrations of inhaled anesthetics to maintain a stable anesthesia. To that end, the minimum anesthetic concentration (MAC) of isoflurane needed to maintain surgical anesthesia was measured. A significantly lower MAC was found after administration of atropine (mean MAC ± sd 2.2 ± 0.3% vs 3.2 ± 0.4%; P < 0.002). Previously, MAC has been indeterminable in chelonians likely due to intracardiac shunting, so this report constitutes the first MAC study performed in a tortoise.

## Introduction

Inhalation anesthesia is routinely used in reptiles^1^, but has been frustratingly difficult to apply in chelonians, because they can hold their breath for extended periods^2^. Even when successfully induced, the anesthetic depth varies considerably despite constant anesthetic gas concentrations. This variability probably explains why the Minimum Anesthetic Concentration (MAC), which is used to determine the percentage of inhalant anesthetics required for surgery^3^ are notably absent for chelonians, whilst it has been determined in both lizards and snakes^1,4–6^. We believe that the variation in anesthetic depths may be a consequence of the unique chelonian cardiovascular anatomy, where cardiac shunts are hypothesized to profoundly influence the absorption rate of anesthetic gases.

As in other non-crocodilian reptiles, chelonians lack complete anatomical separation of the cardiac ventricle, allowing blood to be shunted between the pulmonary and systemic circuits^7–10^. The so-called intracardiac shunts are defined on the basis of their overall direction. The intracardiac right to left (R-L) shunt refers to systemic oxygen-poor blood passing from the right side of the ventricle across the incomplete septum into systemic circulation, thus bypassing the lung, while left to right (L-R) shunting refers to the recirculation of oxygen-rich blood into the pulmonary circulation^8,11^. The direction and magnitude of intracardiac shunt flows are primarily dictated by the balance of the vascular resistances in the systemic and pulmonary vasculature^12^. Chelonians can totally bypass the pulmonary circulation when pulmonary vascular resistance is high in response to increased parasympathetic tone, resulting in a R-L shunt^8,12–14^. Both turtles and tortoises have considerable capacity for cardiac shunting, and the shunt patterns vary consistently with ventilation, such that R-L shunting prevails during apnea, while L-R shunting dominates during ventilation^13,15^.

It was recently observed that snapping turtles (*Chelydra serpentina*) recover significantly faster from isoflurane anesthesia after administration of epinephrine, and it was hypothesized that increased sympathomimetic activity via stimulation of β-adrenergic receptors reduces R-L shunting, providing a more efficient wash-out of isoflurane across the lungs^16^. These studies, however, did not measure pulmonary blood flows and the influence of pulmonary perfusion on anesthetic depth. Atropine, an antagonist to the muscarinic acetylcholine receptors that mediate constriction of the pulmonary circulation at increased parasympathetic tone, has been shown to abolish R-L shunting in chelonians^17,18^. In mammals, including humans, it is well established, both from quantitative models and experimental studies on congenital cardiac malformations or surgical manipulations, that R-L shunts slows the uptake of gas anesthesia^19–21^. The slowing of the uptake of the inhalation anesthesia is inversely proportional to the solubility of the anesthetic, and it would therefore be expected that the uptake of isoflurane should be significantly affected^22^.

In the present study we hypothesized that (i) R-L shunting predominates during isoflurane anesthesia in terrestrial tortoises, but that (ii) atropine administration will elevate pulmonary blood flow, and hence reduce R-L shunting. To investigate these hypotheses, we measured blood flows in the major systemic and pulmonary arteries before and after administration of atropine to evaluate cardiac shunt patterns. To this end we used electrocardiography (ECG) gated magnetic resonance imaging (MRI). We predicted that the elimination of R-L cardiac shunting by atropine administration enables faster distribution of anesthetic gases within the systemic circulation, leading to a deeper and more stable anesthesia. We therefore measured MAC for isoflurane with and without administration of atropine in a cross-over design to address the hypothesis that (iii) MAC is significantly lower after atropine administration.

## Results

**Figure 1:**
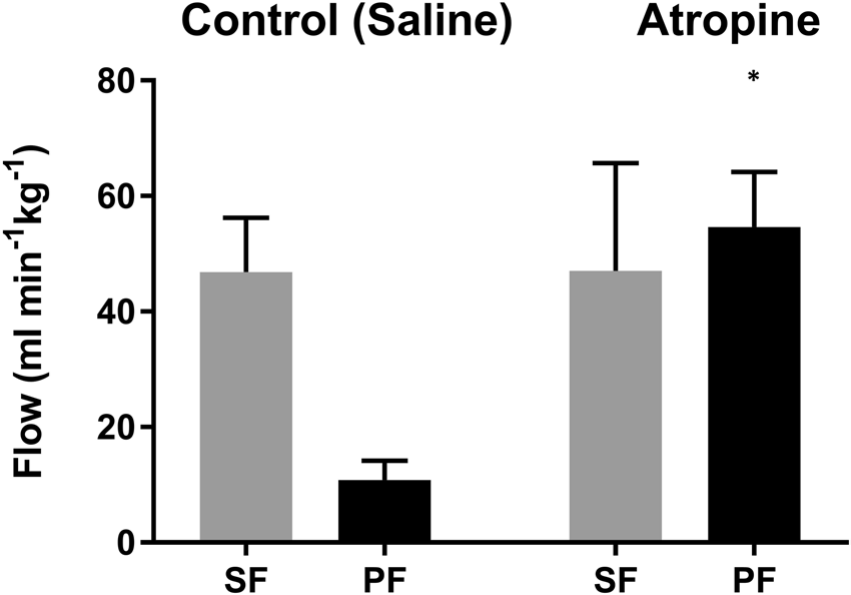
The panel shows systemic (SF, grey) and pulmonary flow (PF, black) (mean ± sd) in five red footed tortoises under isoflurane anesthesia after atropine or saline injection. The pulmonary flow was significantly higher (P < 0.008) with atropine, whereas the systemic flow did not differ. The significant difference (P < 0.05) is indicated with an asterisk (*).

**Figure 2:**
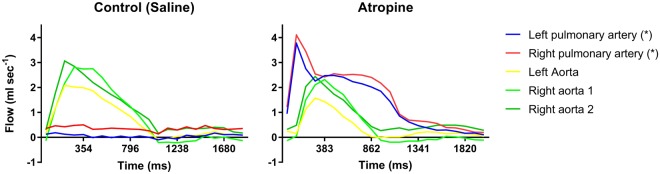
The panel shows flow profiles (mL sec^−1^) in the outflow tract over the cardiac cycle (time in ms), in one individual following injection of saline (left hand panel), and atropine (right hand panel). Vessels shown: left pulmonary artery (blue), right pulmonary artery (red), two branches of the right aorta (light and dark green), and left aorta (yellow). There was a significantly higher flow in the pulmonary arteries after atropine treatment during gas anesthesia. Significant differences (P < 0.05) are indicated with an asterisk (*).

The pulmonary flow was significantly higher (P < 0.008) with atropine, whereas systemic flows were not significantly affected by atropine (Figs 1 and 2). The pulmonary and systemic flows (mean ± sd) were 54.6 ± 9.5 and 47.0 ± 18.7 mL min^−1^ kg^−1^ respectively with atropine, and 10.8 ± 3.4 and 46.8 ± 9.4 mL min^−1^ kg^−1^ respectively with saline. Mean pulmonary and systemic flows were not significantly different from each other under atropine treatment, demonstrating abolition of the R to L shunt, while in 3 out of 5 animals the shunt was reversed. One tortoise was excluded from the MRI study due to inconsistent ECG readings, rendering image interpretation impossible. The intra-observer value between the two flow measurements was 6%. Mean heart rate (HR) did not differ between the two treatments (30 ± 3 and 30 ± 6 bpm in atropine vs saline treatments, respectively).

Atropine significantly lowered MAC in the tortoises (P < 0.002). With atropine the MAC (mean ± sd) was 2.2 ± 0.3%, whereas with saline, MAC was 3.2 ± 0.4%. The maximal time span from injection until the final bracket was reached was 139 minutes. In one tortoise on atropine, a lower bracket could not be determined despite continuously decreasing the end-tidal isoflurane concentration (Et Iso) over 2.4 hours, therefore in that animal, the lowest expiratory isoflurane level with negative response to stimulation was used to calculate the MAC. The HR at the higher and lower brackets did not differ significantly between the two treatments (Higher bracket 45 ± 3 and 46 ± 3 bpm; lower bracket 45 ± 5 and 48 ± 3 bpm with atropine and saline, respectively).

## Discussion

This study demonstrates that R-L cardiac shunting prevails when red-footed tortoises are anesthetized with isoflurane, and that this shunt can be eliminated with atropine. Following elimination of R-L cardiac shunting, it is suggested that pulmonary isoflurane levels more accurately reflect those in the systemic blood circulation, thus animals need lower inhaled concentrations of anesthetic gas to maintain a surgical depth of anesthesia.

Atropine caused a large elevation of pulmonary blood flow, so that the R-L shunt is eliminated upon pharmacological inhibition of the muscarinic receptors on the pulmonary vasculature. It is likely that the presence of the R-L shunt and the low pulmonary blood flow in the control condition slowed the rate of isoflurane anesthesia to an extant where steady-state was not achieved. It has previously been reported that ectotherms with their low metabolism and low rates of tissue perfusion requires considerably longer time to attain steady-state^22^. Thus, upon elimination of the net R-L shunt by atropine, it is likely that the isoflurane levels in the blood were reflective of those in the lungs and hence explain the lower MAC. Our findings are consistent with the slower uptake of gas anesthesia in humans with cardiac malformation, such as tetralogy of Fallot, where R-L shunts prevail, surgical manipulations to induce R-L shunts in experimental animals as well as theoretical considerations of pulmonary gas exchange^19,20,23^.

We suggest that the MAC of 2.2% for isoflurane after atropine administration resembles the “true gas concentration” needed to maintain red-footed tortoises in a surgical level of anesthesia. With the reduction of the cardiac R-L shunt, the anesthetic gas is more effectively distributed within the systemic circulation^21,24^ providing a significant and sizable (31%) reduction in MAC. In contrast, the MAC of the animals that received saline appears erroneously high, because the low pulmonary flow and the R-L shunting restrict isoflurane from reaching the systemic circulation. To our knowledge, no MAC studies in chelonians have been published so far; one co-author tried to determine MAC in red-eared sliders (*Trachemys scripta elegans*), but the study was never completed due to very inconsistent and variable results (Personal communication, Bertelsen). The MAC of isoflurane has been reported to be 1.9 ± 0.6% in radiated rat snakes (*Elaphe radiata)*^5^, 1.8 ± 0.3% to 2.1 ± 0.6% in green iguanas *(Iguana iguana)*^4,6^, and 1.5 ± 0.2% in Dumeril’s monitors *(Varanus dumerilii)*^1^ possibly reflecting the increasing ventricular separation over these phylogenetic groups, ending with varanid lizards that have a pronounced intraventricular flow and pressure separation^9,25,26^. Furthermore, there is evidence that MAC in iguanas decreases over time, likely as a consequence of pulmonary and plasma concentrations reaching equilibrium despite shunting^4^. Temperatures can influence the effective dose of gas anesthesia^27,28^ and the proportion of cardiac shunting in red-eared sliders^18^. The temperature in this study was maintained stable to avoid bias; it is possible that different temperatures may impact the isoflurane MAC and the shunting capacity in red footed tortoises.

Isoflurane anesthesia can increase the vagal tone^29^ and create vasodilation which consequently decreases systemic vascular resistance^30^, both circumstances that could trigger a R-L shunt^8,12–14,31^. Exposure to the gas during induction likely also triggered apnea, a condition that further promotes R-L shunts^15^. In our study, a striking difference in the flow of the pulmonary arteries was detected between the two treatments, with nearly no flow measured in the pulmonary arteries during isoflurane anesthesia without atropine. The reduction of the R-L shunt via atropine was confirmed via ECG gated MRI, representing a novel technique to measure cardiac shunting in chelonians. Although a limited number of animals were used, all showed similar results, strongly suggesting that the results are representative. We believe these results have serious impact on chelonian gas anesthesia, suggesting that where R-L shunting occurs, the full potency of the gas anesthesia is never achieved. We suggest that elimination of the R-L shunt during isoflurane anesthesia via atropine may be a clinically applicable technique, which will allow to maintain a constant, more controllable anesthetic level. Atropine blocks the cholinergic vasoconstriction of the pulmonary circulation and has been shown to eliminate the R-L shunt in red-eared sliders^7,17,18^. Surprisingly, heart rate during isoflurane anesthesia was not affected by atropine, although the pulmonary blood flow clearly was under high parasympathetic tone. It is well established that heart rate is under parasympathetic tone in turtles and other reptiles^8,17,32^, however it is also well-known that anesthesia with pentobarbital almost abolishes this vagal tone^12^, and it would be highly relevant to describe the effects of gas anesthesia on the differential influence on vagal tone on the heart and the pulmonary vasculature in future studies on tortoises. Similarly, studies in aquatic turtles, where R-L shunting may be even more prominent are warranted.

The information about preanesthetic atropine use in reptiles is sparse; some authors advise against the use due to a concern that an increase in salvia viscosity could potentially block the endotracheal tube, but nevertheless suggest using it during bradycardia^6^. A very high atropine dose was used in this study to eliminate the R-L shunt during isoflurane anesthesia, but no adverse effects were observed. Further research determining optimal dosing and assessing potential untoward effects of atropine is, however, required.

In conclusion, R-L intracardiac shunting predominates during isoflurane anesthesia in the red-footed tortoise. ECG-gated MRI served as a useful, novel tool to study arterial flow and shunt physiology during gas anesthesia in reptiles. This state-of the art imaging technology confirmed the vascular response after atropine treatment and the consequent elimination of the R-L shunt. Due to elimination of shunting, passage of anesthetic gas into the systemic circulation was facilitated, making gas anesthesia more controllable and stable, reducing the MAC significantly.

## Methods

The study was approved by the Danish Experimental Animal Inspectorate (permit # 2015-15-0201-00684), all experiments were performed in accordance with relevant guidelines and regulations. Six adult female red-footed tortoises (*Chelonoidis carbonaria*) with a mass (mean ± sd) of 3.3 ± 0.8 kg were housed together at Aarhus University in an approximately six square meter indoor enclosure, equipped with hiding places, UV lights (160 W UVA + B, Exoterra, Denmark), and heat lamps providing a temperature gradient in the enclosure. The room temperature was maintained at 26–28 °C with 60–80% humidity and a 12:12 light: dark cycle. The tortoises had access to water at all times and were fed mixed vegetables daily and a higher protein source, such as fresh dead mice on a weekly basis. Food was withheld 12 h prior to anesthesia.

Prior to the study, animals were weighed and blood was collected from the jugular vein to perform a differential count and a biochemical profile (Abaxis VetScan VS 2, scil animal care company GmbH, 68519 Viernheim, Germany), and to determine the hematocrit. The animals were deemed healthy on the basis of a general clinical examination, their constant or increasing mass and the blood results.

### MAC study

In a randomized cross-over study, each animal was anesthetized twice, once receiving saline and once receiving atropine, with a minimal washout period of five days. The animals were chamber induced 49 ± 31 min with isoflurane (Vetflurane, VIRBAC, 06516 Carros, France; 4–5 mL of Isoflurane in 50 L chamber) until the animals reached a sufficient level of anesthesia for intubation. After administration of 0.05 mL of lidocain (Xylocaine 20 mg/ml AstraZeneca (SE)) topically on the glottis, animals were intubated with an uncuffed tube of appropriate size, ranging from 2.5 to 3.5 mm internal diameter. Following intubation, animals were maintained on isoflurane using a semiclosed circle anesthetic system (Hallowell Small Animal AWS, Pittsfield, MA 01201) with an agent-specific vaporizer (Ventilator Tec 3, Iso Tec, Ohmeda GR Healthcare, Liverpool UK). The animals were mechanically ventilated at four breaths a minute with a tidal volume of approximately 12.5 ml/kg. Within ten minutes after intubation the animal was injected with either 1 mg/kg atropine (Atropine sulfate in 0.9% saline, Sigma Aldrich, DE 4 mg/mL) or with the same volume of saline (0.9% sodium chloride Fresnius Kabi SE) intravenously in the jugular vein. Both the order of the individuals and the treatment were randomized via a randomizing internet platform (www.random.org).

Following intubation, at five minute intervals, the end-tidal isoflurane concentration (Et Iso) (IRMA AX+, Masimo, Sweden), the body temperature (BT) via a thermometric probe, the respiration rate (RR) and quality via the capnography waveform (IRMA AX +, Sweden; Cardell touch monitor, Midmark, USA) and the heart rate via Doppler (Nicolet Elite Doppler, 5 MHz probe, Natus Medical, Inc. Pleasanton, CA 94566 USA) were recorded. A target BT of 30 °C was maintained using an electric heating pad (Melissa electric heating pad 631-015; Adexi A/S, Denmark) so that the variation in BT between the two treatments of each individual tortoise did not exceed ±1 °C.

MAC was determined via a bracketing technique^33^, starting 60 ± 3 minutes (mean ± sd) after the injection of atropine or saline and after the Et Iso was stable for 15 minutes. One interdigital region was pinched with an arterial clamp for 60 seconds. The reaction of the pinch was evaluated as positive if the animal retracted the stimulated limb in a coordinated fashion, moved the contralateral limb, or moved other parts of the body. If there was a response to stimulation, the isoflurane concentration was increased by 10–20% (Mean ± sd = 12 ± 7%). If there was no response, the isoflurane concentration was decreased by 10–20% (Mean ± sd = 13 ± 2%). Stimulation was repeated after the new isoflurane level had remained stable for 15 minutes. This technique was continued until the opposite reaction to that for the first stimulation was triggered. The MAC for each tortoise was then calculated as the mean of the highest Et Iso that allowed movement and the lowest concentration that did not. The starting concentration of Et Iso of each animal depended on calculated MAC of the previous animal undergoing the same treatment.

### Anaesthesia for MRI Scanning

This part of the study was carried out using the same tortoises, equipment, induction protocol and washout periods but following new randomization. The only difference in set up was that longer tubing was required to connect the endotracheal tube because the ventilator needed to be placed outside the MRI room. The long tubes were pre-filled with 5% isoflurane before connecting them to the intubated tortoise. A very high concentration of 5% isoflurane was maintained during scanning. This was done to exacerbate the possible shunting and to prohibit any movement of the animal. Close monitoring of the tortoise including Et Iso during the MRI was not technically possible. Chamber induction (54 ± 9 min) was followed by intubation, and the injection of atropine or saline. Scanning commenced between 30 and 70 minutes (43 ± 12 min) after injection, and scanning time was between 11 and 39 minutes (18 ± 8 min). The tortoises were mechanically ventilated as previously described, and maintained at a vaporizer setting of 5% isoflurane during the entire procedure. Throughout scanning, the HR was monitored with the four lead electrocardiogram (ECG) of the MRI scanner collected via non-metallic clinical attachments on the four limbs and the tortoises were heated via a digital feedback warm air blower (SAII, Stony Brook, NY 11790, USA). The BT difference between the two scans of one individual, calculated as the mean before and after scanning, ranged from 0 to 2.8 °C (1.2 ± 1.4 °C, mean ± sd).

### MRI and flow determination

**Figure 3:**
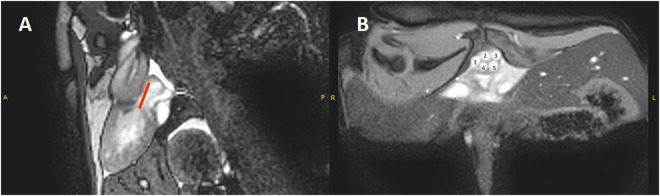
(**A**) The cardiac landmark for flow determination of the long axis view at its peak systole in the magnetic resonance image. The measured distance (red line) from the valve to the cross section slice was used for the flow measurements. (**B**) Cross section of the tortoise and of the cardiac outflow tract used for flow determination. In this animal, the vessels for flow determinations used were the right aorta which is already branched (1, 4), the left pulmonary artery (3), left aorta (2) and the right pulmonary artery (5).

A clinical 1.5 T MRI scanner (Philips Healthcare, 1096 BC Amsterdam, The Netherlands) was used to visualize the heart and to perform the flow measurements. A clinical head coil was used as receiver. After initial scout imaging for anatomical positioning, a number of long axis views of the heart was acquired. An ECG gated Balanced Steady-State-Free-Precession sequence was used, slice thickness was 4 mm and pixel size was 1.0 × 1.0 mm. Flow measurements were acquired in 7 slices in the outflow tract orthogonal to the flow direction using a phase contrast flow sequence. Slice thickness was 4 mm, pixel size 0.8 × 0.8 mm and number of cardiac frames was 27. The velocity sensitivity parameter was 50 cm/s. Flow was analyzed using in-house developed software (Siswin, Aarhus, Denmark). Two independent, blinded observers identified the right and left pulmonary arteries and either the right and left aortas or further aortic branching into three or four vessels (Fig. 3). Slice selection was taken as the first slice above the division of the common pulmonary artery into left and right pulmonary arteries, where the circular cross-sectional appearance of the outflow tract showed that slice was orthogonal to flow. Long axis views were used to confirm the distance from the aortic valves at the maximum ventricular contraction to the slice used for flow measurement (Fig. 3), to ensure the atropine and saline group slice selection was comparable (Mean ± sd distance 7.62 ± 1.79 mm saline group, 7.76 ± 1.5 mm atropine group). The observers adjusted the vessel marker and calculated the flow for each heart phase through the entire heart cycle for all vessels. The sum of the branching aortas and the two pulmonary arteries were used for statistical analysis. An intra-observer value of the differences of the measurements of less than 10% were regarded as acceptable to justify the use of this method of flow determination. The mean of the two observers’ measurements were used for flow calculations.

### Statistical analyses

were performed using GraphPad Prism version seven for Windows (GraphPad Software, Inc. La Jolla CA 92037, USA), using the Mann-Whitney test to compare atropine and saline injected individuals. The MACs, and additionally the HR between the upper and lower brackets of the two different treatments, were compared. In the MRI study, the pulmonary and systemic flows and the HR were compared between atropine and saline injected individuals. Differences were considered significant at values of P < 0.05.
